# The effects of protective lung ventilation on regional cerebral oxygen saturation in intracranial tumor operation during dura opening: study protocol for a randomized controlled trial

**DOI:** 10.1186/s13063-019-4025-9

**Published:** 2020-02-07

**Authors:** Jinlu Li, Xuemei Wu, Hairui Liu, Ying Huang, Yueqin Liu, Hong Xie, Jun Dong

**Affiliations:** 1 Department of Anesthesiology, The Second Affiliated Hospital of Soochow University, 1055 Sanxiang Road, Suzhou, 215004 Jiangsu China; 2 Department of Neurosurgery, The Second Affiliated Hospital of Soochow University, 1055 Sanxiang Road, Suzhou, 215004 Jiangsu China

**Keywords:** Randomized controlled trial, Regional cerebral oxygen saturation, Lung ultrasound, Brain relaxation, Craniotomy

## Abstract

**Objective:**

The objective of this trial is to investigate the effects of protective lung ventilation on regional cerebral oxygen saturation (rSO_2_) during dura opening, that is from Ta (after dura opening) to Tb (before dura closing), in patients undergoing intracranial tumor surgery.

**Methods:**

This is a randomized controlled trial which will be carried out at the Second Affiliated Hospital of Soochow University. Fifty-four patients undergoing intracranial tumor surgery will be randomly allocated to the control group (C group) or the protective lung ventilation group (P group). In the C group, the tidal volume (VT) will be set at 8 ml/kg of predicted body weight, but positive end-expiratory pressure (PEEP) and recruitment maneuvers will not be used. In the P group, VT will be set at 6 ml/kg of predicted body weight combined with individualized PEEP during dura opening, while in other periods of general anesthesia, VT will be set at 8 ml/kg of predicted body weight. The level of rSO_2_, partial pressures of oxygen and carbon dioxide, oxygenation index, lactic acid level in arterial blood, and mean arterial pressure will be compared before anesthesia (T0), before dura opening (T1), after dura closing (T2), and 24 h after surgery (T3). Lung ultrasound scores will be measured at T0 and T3. The degree of brain relaxation at T1 and T2 will be evaluated by the surgeon using the brain relaxation scale. The amount of vasoactive drugs used and blood loss will be recorded during surgery. The duration of operation and reoperation rate will be recorded. The primary outcome of this study is the changes in rSO_2_ within 24 h postoperatively.

**Discussion:**

This study aims to determine whether protective lung ventilation during dura opening can improve rSO_2_ and the state of pulmonary ventilation in patients undergoing intracranial tumor surgery, and to investigate whether this strategy affects the degree of brain tissue swelling and the reoperation rate after operation. If our results are positive, this study will show that protective lung ventilation during dura opening can be used effectively and safely in neurosurgical patients undergoing craniotomy for tumor resection.

**Trial registration:**

Chinese Clinical Trial Registry, ChiCTR1900025632. Registered on 3 September 2019. chictr.org.cn.

## Background

With the development of society and the progress of science and technology, more and more people receive delicate and complex procedures such as neurosurgery. Almost all these patients are under general anesthesia, which is basically inseparable from mechanical ventilation. Of these patients, 15–20% have different degrees of alveolar collapse at the bottom of the lung before operation, and this phenomenon could persist for several days after operation due to the influence of mechanical ventilation with endotracheal intubation. Pulmonary complications play an important role in death and disability in patients who have has general anesthesia [1–3]. Craniotomy always requires the patient to be under general anesthesia for a long time with prolonged mechanical ventilation, which leads to a higher risk of postoperative atelectasis and pulmonary infection [4, 5]. Atelectasis and pulmonary infection can seriously affect pulmonary ventilation and even lead to severe hypoxemia. Moreover, the long time period of the brain operation is more likely to cause an imbalance of brain oxygen supply and consumption. This imbalance of brain oxygen supply and consumption may lead to deterioration of brain function, such as postoperative cognitive function [6]. Postoperative cognitive dysfunction (POCD) will lower the quality of life, increase mortality, and aggravate the financial and mental burden of patients.

Protective lung ventilation (PLV) strategies have been recognized by many anesthesiologists and are widely used in clinical anesthesia [7, 8]. Relevant studies suggest that low tidal volume combined with positive end-expiratory pressure (PEEP) ventilation and alveolar recruitment maneuver (ARM) is the most widely used PLV strategy, and it can reduce lung volume damage and pulmonary barotrauma, improve pulmonary function, and decrease postoperative pulmonary complications [9]. Theoretically, low tidal volume prevents excessive alveolar expansion [10], and higher PEEP prevents pulmonary atelectasis [11]. However, gradually increased PEEP to the level of 20 cm H_2_O or even higher is often needed in traditional PLV strategies [5, 9] which will obviously affect the circulation and intracranial pressure (ICP) of patients [12] and may increase airway pressure and reduce cerebral venous reflux and intraoperative operating space, thus limiting its application in patients with craniotomy. In addition, anesthesiologists often use a single PEEP or pulmonary retention mode, ignoring individual differences among patients, thus affecting the effect of PLV [13, 14].

In recent years, with the development of medical monitoring equipment, regional cerebral oxygen saturation (rSO_2_) monitoring technology [15, 16] has been gradually developed and used in clinical anesthesia. It provides a condition for real-time monitoring of the perfusion level of brain tissue in patients undergoing craniotomy and provides technical support for carrying out clinical research on protective pulmonary ventilation during craniotomy.

Near infrared spectrometry (NIRS) using near infrared technology is similar to pulse oxygen monitoring, which is commonly used. Near infrared light with a wavelength of 650–1100 nm has a good penetrability to human tissues such as scalp, skull, and brain, even up to several centimeters. The major color base (hemoglobin, Hb) attenuated in the intracranial area of NIR light results in changes in the light intensity of penetrating human tissues. The oxygenation of brain tissues is evaluated by measuring the changes in the absorption spectrum, which are accompanied with changes in oxygenation state [17].

Up to the present, in consideration of the risks associated with PEEP and recruitment maneuvers, there are no correlative randomized controlled trials to explore the efficacy and safety of intraoperative pulmonary protective ventilation strategies in patients undergoing craniotomy. However, due to the disappearance of ICP after the dura opening during craniotomy, an individualized protective pulmonary ventilation strategy may avoid adverse effects on cerebral perfusion. The purpose of this study is to evaluate the effects of PLV strategies with individualized PEEP during dura opening on rSO_2_ in patients undergoing intracranial tumor surgery. Other outcomes include intraoperative brain relaxation, lung ultrasound scores 24 h after surgery, the reoperation rate within 1 week after operation, the amount of blood loss, and dosage of vasoactive drugs during surgery.

## Methods

### Study design

**Fig. 1:**
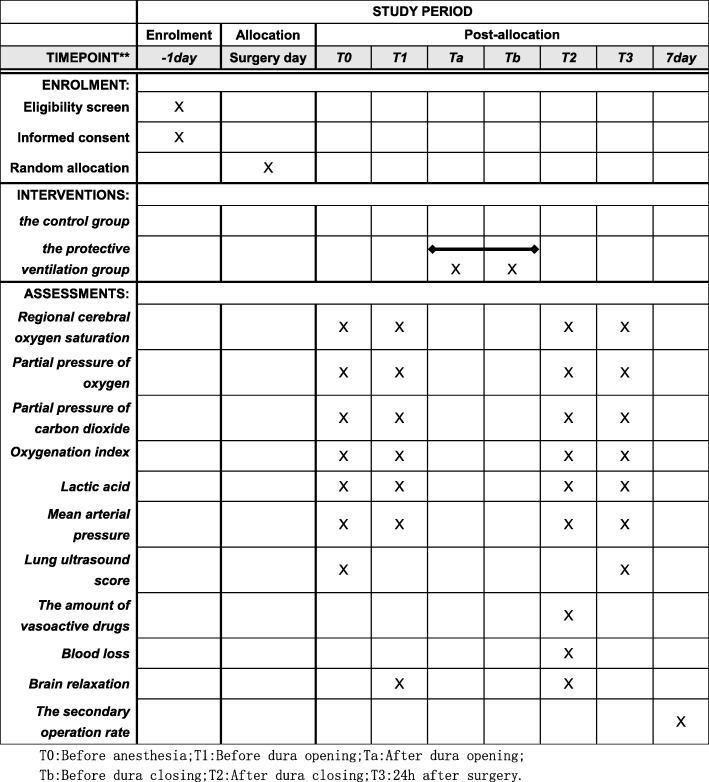
Schedule of enrollment, interventions, and assessments

This is a single-center, randomized controlled trial which is being conducted at the Second Affiliated Hospital of Soochow University. Recruitment began on 3 September 2019. All patients eligible for inclusion will be recruited continuously until recruitment completion. The schedule of enrollment, interventions, and assessments is shown in Fig. 1. The basic information of patients will be as shown in Table 1. The Standard Protocol Items: Recommendations for Interventional Trials (SPIRIT) checklist is provided as Additional file 1.

**Table 1:** Patient characteristics and baseline data

Characteristic	C group	P group
Male/female		
Age, years		
Body mass index (BMI), kg/m^2^		
Predicted body weight, kg		
American Society of Anesthesiologists (ASA) physical status, I/II		

Patients will be randomized after signed informed consent and confirmation of inclusion and exclusion criteria. Randomization will be conducted via a computer-generated age stratification randomized controlled table. Age stratification is according to (1) 18 < younger ≤ 40 years; (2) 40 < elder ≤ 65 years. Patients who meet the enrollment criteria will be randomly allocated to the control group (C group) or the PLV group (P group) within 24 h before surgery. The allocation ratio is 1:1. Permuted randomization will be used and stratified by age. The designated staff will perform the allocation sequence. The designated staff assistants will assign participants to the interventions. This research staff will implement the allocation sequence through sealed, opaque, and stapled envelopes. Corresponding envelopes will not be opened until the enrolled participants complete the trial. The anesthesiologist who is responsible for the anesthesia implementation will know the grouping but will not participate in the follow-up visit. However, the neurosurgeon who evaluates brain relaxation will be blinded to the group allocation. The patients and the outcome assessor are all blinded to the grouping.

### Selection and withdrawal of participants

#### Recruitment

Participants will be recruited from the neurosurgical wards and identified by their presence on surgical lists. The investigator informs the participant or the participant’s legal representative of all aspects. The study intervention will be completed immediately after the surgery, but follow-up visits will extend to 1 week after surgery. The medical records will be reviewed following hospital discharge for in-hospital complications and medication usage.

#### Inclusion criteria

Patients will be included if they fulfill all of the following criteria:

1. Patients scheduled to receive elective intracranial primary tumor resection who are aged between 18 and 65 years old
2. The maximum diameter of the tumor is 2–5 cm (magnetic resonance imaging, MRI)
3. American Society of Anesthesiologists (ASA) classification I–II
4. 18.5 < body mass index (BMI) < 28
5. Glasgow Coma Scale score of more than 8 points.

#### Exclusion criteria

Patients will be excluded if they have any of the following criteria:

1. Patients with chronic lung disease, pulmonary infection, or other severe pulmonary complications such as acute respiratory failure
2. Patients with a history of pulmonary surgery
3. Patients with severe brain, heart, liver, or kidney diseases
4. Patients with nerve injury affecting preoperative breathing
5. Pregnant women
6. Those who refuse to participate in the research.

#### Termination criteria

The following will cause participants to be terminated from the study:

1. Duration of anesthesia < 4 h or > 8 h, or duration of operation < 2 h or > 6 h
2. Patients with significantly increased intraoperative ICP or swelling of brain tissue
3. Patients with intraoperative endotracheal catheter after surgery
4. Repeat intubation or operation within 24 h after operation.

### Study intervention

#### Related parameter setting during operation

All patients will be randomly allocated to the C group or the P group according to the computer-generated random number table. In the C group, tidal volume (VT) will be set at 8 ml/kg of predicted body weight, with PEEP = 0, and recruitment maneuvers will not be used. The predicted body weight is calculated as follows: for men, 50 + 0.91 (height in centimeters – 152.4); and for women, 45.5 + 0.91 (height in centimeters – 152.4) [18]. In the P group, VT will be set at 6 ml/kg of predicted body weight combined with individualized PEEP during intraoperative dura mater opening [6, 7], but in other periods of general anesthesia, VT will be set at 8 ml/kg of predicted body weight. For the titration method of individualized PEEP [19], VT and respiratory rate will be fixed at 6 ml/kg and 15 beats per minute during PEEP trial. Titration can only begin once the dura is opened. The titration for the individual PEEP can then be initiated by increasing PEEP from 0 to 10 cm H_2_O incrementally. Each PEEP level (0, 1, 2, 3, 4, 5, 6, 7, 8, 9, 10 cmH_2_O) will be maintained for 1 min, and the pulmonary compliance of the last cycle will be recorded at each PEEP level. The PEEP value at the highest compliance will then be selected as the individual PEEP of the patient.

Peripheral venous access will be established after the patient enters the operating room. If necessary, central venous access will be established. Noninvasive blood pressure (NBP), electrocardiogram (ECG), heart rate (HR), oxygen saturation (SpO_2_), and bispectral index (BIS) will be routinely monitored. Radial artery catheterization under local anesthesia will be used to monitor invasive arterial pressure and collect blood samples. All of the above data will be collected completely. Fentanyl 5 μg/kg, etomidate 0.3 mg/kg, rocuronium 0.6 mg/kg will be used for induction and will be started after oxygen flow of 0.1 L/kg/min has been given by mask for 2 min. Volume-controlled mechanical ventilation will be conducted with a Primus anesthesia machine (Dräger, Lübeck, Germany) after the endotracheal catheter is inserted to a correct position. The VT will be set at 8 ml/kg of predicted body weight, the inhalation oxygen fraction (FiO_2_) will be set at 0.5, the inhalation-expiration ratio (I:E) = 1:2, and the fresh gas flow will be set at 1 L/min. The respiration rate will be adjusted according to the result of end-expiratory carbon dioxide (ETCO_2_), and the end-tidal CO_2_ pressure (PetCO_2_) will be maintained between 30 and 35 mmHg. There will be 1% sevoflurane combined with propofol and remifentanil to maintain anesthesia, and the BIS value will be maintained at 45–55. During the operation, intermittent injection of fentanyl and rocuronium will be used to deepen the anesthesia. Extubation indications are that patients are awake and cooperating and muscle relaxation monitoring train-of-four (TOF) stimulation > 90% [20]. Intraoperative fluid intake and urine volume will be monitored closely. The level of rSO_2_ will be recorded in the tumor surgery area of the patients before anesthesia (T0), before dura opening (T1), after dura closing (T2), and 24 h after surgery (T3). Arterial blood of the patients will be collected for blood gas analysis. Lung ultrasound scores (LUSs) will be performed at T0 and T3. The degree of brain relaxation at T1 and T2 will be evaluated by the surgeon using the brain relaxation scale. The amounts of vasoactive drugs used and blood loss will be recorded during surgery. The duration of operation and reoperation rate will be recorded.

### Study objective

#### Primary and secondary outcomes

The primary outcome of this study is the changes in rSO_2_ within 24 h postoperatively. See Table 2.

The secondary outcomes are as follows (the corresponding parameters are listed in Tables 2 and 3):

1. Changes between preoperative and postoperative LUSs in patients. LUSs will be used to evaluate postoperative atelectasis. The patient’s chest is divided into 12 quadrants. Each of the 12 quadrants is assigned a score of 0 to 3 according to a modified grading system (Table 4). The LUS score (0–36) is then calculated by adding up the 12 individual quadrant scores, with higher scores indicating more severe atelectasis [21].
2. The mean arterial pressure changes during intraoperative pulmonary protective ventilation.
3. The partial pressures of oxygen (PaO_2_) and carbon dioxide (PaCO_2_), oxygenation index (OI), and lactic acid level (Lac) in arterial blood changes during intraoperative pulmonary protective ventilation.
4. The amount of vasoactive drugs and blood loss will be compared in the two groups during surgery.
5. Intraoperative brain relaxation, which will be scored by the neurosurgeons after opening the cranium and before opening the dura. They will use a 4-point scale [22]: 1, completely relaxed; 2, satisfactorily relaxed; 3, firm brain; 4, bulging brain.
6. The secondary operation rate in 1 week after surgery (reoperation rate).

**Table 2:** Comparison of rSO_2_, PaO_2_, PaCO_2_, OI, Lac, and MAP

		T0	T1	T2	T3
rSO_2_	C group				
	P group				
PaO_2_	C group				
	P group				
PaCO_2_	C group				
	P group				
OI	C group				
	P group				
Lac	C group				
	P group				
MAP	C group				
	P group				

*T0* before anesthesia, *T1* before dura opening, *T2* after dura closing, *T3* 24 h after surgery, *rSO*_*2*_ regional cerebral oxygen saturation, *PaO*_*2*_ partial pressure of oxygen, *PaCO*_*2*_ partial pressure of carbon dioxide, *OI* oxygenation index, *Lac* lactic acid level, *MAP* mean arterial pressure

**Table 3:** Perioperative parameters

	C group	P group
Tidal volume, ml		
Individual PEEP, cmH_2_O		
Duration of anesthesia, min		
Duration of operation, min		
Duration of dura opening, min		
Tumor size (maximum diameter)		
Brain relaxation scale (T1)		
Brain relaxation scale (T2)		
Lung ultrasound score (T0)		
Lung ultrasound score (T3)		
Volume of total fluid, ml		
Amount of bleeding, ml		
Volume of urine, ml		
Dosage of vasoactive agent, mg		
Reoperation rates

**Table 4:** Modified lung ultrasound scores

Quotation	Normal aeration	Small loss of aeration	Moderate loss of aeration	Severe loss of aeration
	0	1	2	3
Modified lung ultrasound score	0–2 B lines	≥ 3 B lines OR 1 or multiple small subpleural consolidations separated by a normal pleural line	Multiple coalescent B lines OR multiple small subpleural consolidations separated by a thickened or irregular pleural line	Consolidation OR small subpleural consolidation of > 1 × 2 cm in diameter

Lung ultrasound scores can be calculated by adding up the 12 individual pulmonary quadrant scores yielding a score between 0 (no aeration loss) and 36 (complete aeration loss)

### Reporting of adverse events

All adverse events will be recorded and closely monitored until resolution or stabilization. In the event of any serious adverse event (≥ grade 3) [23], the event will be immediately reported to the Endpoint Adjudication Committee, which will determine the severity and causality of the adverse events. The chief investigator will be responsible for all adverse event reporting.

### Withdrawal from the trial

We will consider patient withdrawal from the trial if the following conditions occur: (1) severe brain swelling during the operation; (2) the patient has a cough during surgery; (3) the patient has persistent hypotension and circulatory instability.

### Data collection and management

All the patient information will be obtained through the electronic medical record system. The consent of the treating neurosurgeon, who will help us make the neurological diagnosis, will also be obtained. All personal information will be collected through the hospitalized medical records by a member of the research team and be kept strictly confidential for research purposes only. The research team members will be responsible for maintaining personal data. Only the primary investigator and the designated researcher can obtain interim results and final test data.

#### Data Monitoring Committee

The project will be monitored by a Data Monitoring Committee (DMC) composed of specialists in anesthesiology, ethics, statistics, and methodology. The DMC will audit through regular interviews or telephone calls.

### Sample size and justification

We calculated the sample size through the website http://www.sample-size.net/sample-size-proportions/.

The difference in brain oxygen saturation before and after surgery was 3.6 ± 4.1, α = 0.05, β = 0.2 [7]. Based on this, it can be calculated that the sample size required for our study is 44 cases. Considering a 20% shedding rate, a total of 53 participants (44 + 44*20%) need to be recruited. Due to the 1:1 distribution ratio, a total of 54 cases will be recruited.

### Statistics

The SPSS 19.0 software package for Windows (SPSS, Inc., Chicago, IL, USA) will be used for all statistical analyses. The quantitative variables will be expressed as mean ± standard deviation (SD) or median (interquartile range [IQR]) and analyzed by using the analysis of variance (ANOVA) or Mann-Whitney *U* test. Multiple comparisons among continuous variables at different time points will be performed using the Student-Newman-Keuls method for post hoc test. The incidence of reoperation rate will be expressed as the number of patients (percentage) and be analyzed by using the chi-square (χ^2^) test. *P* values < 0.05 will be considered to be statistically significant. After the follow-up of half of the cases, the interim analysis will be conducted to evaluate the validity of the main results.

## Discussion

This study is a single-center, randomized controlled trial exploring whether protective lung ventilation (PLV) during intraoperative dura opening can improve regional cerebral oxygen saturation (rSO_2_) in neurosurgical patients.

The incidence of postoperative pulmonary complications (PPCs) is high due to the long mechanical ventilation in neurosurgery. Qaseem et al. [24] reported that the risk of PPCs increased when the operation time is more than 4 h. The incidence of PPCs was 28.4% (20.2–37.9%) in patients with neurosurgery lasting for longer than 300 min [25]. PEEP can lower the incidence of postoperative respiratory complications, prevent atelectasis, and reduce the risk of ventilators associated with lung injury.

In this study, individual PEEP (< 10cmH_2_O) will be used to avoid the effect of high PEEP on intracranial pressure (ICP). It is a crucial issue that PEEP can be safely used in craniotomy. Therefore, pulmonary protective ventilation will be performed during dura opening, and cerebral relaxation will be assessed before dura incision. If the ICP is elevated enough to affect the operation for using PEEP, we will abandon the case and change the ventilation parameters. The case will be reported to the principal investigator.

The rSO_2_ is actually the mixed oxygen saturation of local brain tissues, which can better reflect the change of brain oxygen supply and consumption balance during the perioperative period. Samra et al. [26] studied 100 patients who underwent carotid endarterectomy. They found that if the rSO_2_ value decreased by 20% compared with the baseline value after internal carotid artery occlusion, it predicted the possibility of neurological complications, and indicated that its sensitivity was 80% and its specificity was 82%. Since the ratio of cerebral blood volume to arterial/venous blood flow is approximately 20:80, the NIRS value mainly represents cerebral venous oxygen saturation, which is completely unaffected by hypoxemia and hypocarbonemia, and better reflects the changes in the balance of oxygen supply and consumption in the brain [27]. Near infrared spectroscopy (NIRS) as a brain oxygen monitoring method has the following characteristics: it is continuous and noninvasive and convenient, and it has a high degree of sensitivity and specificity [28]. Monitoring rSO_2_ can detect changes of the cerebral blood flow and oxygen supply and consumption balance in the brain area as early as possible, and judge the degree of cerebral ischemia and hypoxia and changes in brain function. Timely adjustment of the anesthesia plan is helpful to guide perioperative anesthesia management, so as to prevent POCD, shorten the hospitalization period, and improve quality of life.

We focus on whether a pulmonary protective ventilation strategy can affect cerebral venous reflux and brain tissue oxygenation and, ultimately, the prognosis of patients. PLV after incision of the dura can reduce the returned blood volume that results in exposing potential bleeding spots, which is beneficial in helping the surgeon to stop bleeding. Due to the opening of the dura, the ICP disappears, and the decreased cerebral perfusion pressure caused by the expansion of the lung is improved.

This study is a prospective, randomized controlled trial. This study aims to investigate the effect of intraoperative pulmonary protective ventilation in neurosurgical craniotomy. If we are able to demonstrate the safety and effectiveness of intraoperative pulmonary protective ventilation with individualized PEEP during dura opening in neurosurgical craniotomy, it will improve the prognosis of patients undergoing neurosurgery and reduce medical costs.

### Trial status

The study was registered on the registry website http://chictr.org.cn/ with registration number ChiCTR1900025632 on 3 September 2019. The protocol version is 3.0, dated 3/9/2019. The study began on 3 September 2019, and the planned completion date will be September 2020. The trial status is currently recruiting. Recruitment began on 3 September 2019, and the planned recruitment completion date will be June 2020.

## Supplementary information

**Additional file 1.** SPIRIT 2013 checklist: recommended items to address in a clinical trial protocol and related documents.

## Data Availability

The datasets analyzed during the current study are available from the corresponding author on reasonable request.

## Abbreviations

ARM: Alveolar recruitment maneuver
BIS: Bispectral index
ECG: Electrocardiogram
ETCO_2_: End-expiratory carbon dioxide
FiO_2_: Oxygen fraction
HR: Heart rate
ICP: Intracranial pressure
Lac: Lactic acid level
MAP: Mean arterial pressure
NBP: Noninvasive blood pressure
NIRS: Near infrared spectrometer (spectrometry, spectroscopy)
OI: Oxygenation index
PaCO_2_: Partial pressure of carbon dioxide
PaO_2_: Partial pressure of oxygen
PEEP: Positive end-expiratory pressure
PetCO_2_: End-tidal CO_2_ pressure
PLV: Protective lung ventilation
PPC: Postoperative pulmonary complication
rSO_2_: Regional cerebral oxygen saturation
SpO_2_: Oxygen saturation
VT: Tidal volume
